# An Intrinsically Disordered Region of the Adenovirus Capsid Is Implicated in Neutralization by Human Alpha Defensin 5

**DOI:** 10.1371/journal.pone.0061571

**Published:** 2013-04-19

**Authors:** Justin W. Flatt, Robert Kim, Jason G. Smith, Glen R. Nemerow, Phoebe L. Stewart

**Affiliations:** 1 Department of Pharmacology and Cleveland Center for Membrane and Structural Biology, Case Western Reserve University, Cleveland, Ohio, United States of America; 2 Department of Molecular Physiology and Biophysics, School of Medicine, Vanderbilt University, Nashville, Tennessee, United States of America; 3 Department of Microbiology, University of Washington, Seattle, Washington, United States of America; 4 Department of Immunology and Microbial Science, The Scripps Research Institute, La Jolla, California, United States of America; French National Centre for Scientific Research, France

## Abstract

Human α-defensins are proteins of the innate immune system that suppress viral and bacterial infections by multiple mechanisms including membrane disruption. For viruses that lack envelopes, such as human adenovirus (HAdV), other, less well defined, mechanisms must be involved. A previous structural study on the interaction of an α-defensin, human α-defensin 5 (HD5), with HAdV led to a proposed mechanism in which HD5 stabilizes the vertex region of the capsid and blocks uncoating steps required for infectivity. Studies with virus chimeras comprised of capsid proteins from sensitive and resistant serotypes supported this model. To further characterize the critical binding site, we determined subnanometer resolution cryo-electron microscopy (cryoEM) structures of HD5 complexed with both neutralization-sensitive and -resistant HAdV chimeras. Models were built for the vertex regions of these chimeras with monomeric and dimeric forms of HD5 in various initial orientations. CryoEM guided molecular dynamics flexible fitting (MDFF) was used to restrain the majority of the vertex model in well-defined cryoEM density. The RGD-containing penton base loops of both the sensitive and resistant virus chimeras are predicted to be intrinsically disordered, and little cryoEM density is observed for them. In simulations these loops from the sensitive virus chimera, interact with HD5, bridge the penton base and fiber proteins, and provides significant stabilization with a three-fold increase in the intermolecular nonbonded interactions of the vertex complex. In the case of the resistant virus chimera, simulations revealed fewer bridging interactions and reduced stabilization by HD5. This study implicates a key dynamic region in mediating a stabilizing interaction between a viral capsid and a protein of the innate immune system with potent anti-viral activity.

## Introduction

Human α-defensins are small (3–5 kDa), positively charged, amphipathic, naturally occurring peptides that are abundant in neutrophils and Paneth cells of the small intestine [1]. Structure determination of these molecules revealed that they have a three-stranded beta-sheet fold stabilized by disulfide bonds and can readily form dimeric complexes [2]–[4]. New functional studies showed the importance of dimerization for α-defensin mediated inactivation of both bacteria [5] and viruses [6], and a structural study of membrane-bound α-defensin supports a dimer pore mechanism for membrane disruption [7]. Humans express six α-defensins (HNP1–4, HD5, and HD6) and multiple β-defensins that are distinguished by the arrangement of their disulfide bonds and their expression patterns. Of the six α-defensins, HD6 forms an atypical dimer that undergoes further ordered self-assembly to form fibrils that entangle bacteria [8].

Currently, there is little structural information on the recognition of microbial agents by defensins. The antibacterial activities of defensins against both Gram-positive and Gram-negative organisms have been characterized with the major bactericidal mechanism involving membrane disruption, although other mechanisms have been recently proposed [9]–[11]. An understanding of the antiviral properties of defensins is beginning to emerge [6], [12], [13]. For enveloped viruses, defensins can suppress viral infection by direct inactivation of the virion via membrane disruption [12], interference with viral membrane fusion [12], [14] and by modulation of immunity and other biological responses of the host [15]. Viruses that lack envelopes including human adenovirus (HAdV), human papillomavirus (HPV), and polyomaviruses are neutralized by α-defensins despite the absence of a lipid membrane target [16]–[21].

A previous structural and functional characterization of α-defensin neutralization of HAdV showed that the mechanism of inactivation is species specific and dependent upon α-defensin tertiary structure [13]. A cryoEM structural analysis of HD5 complexed with a neutralization-sensitive HAdV chimera led to a model for neutralization in which HD5 binds at a point of contact between the vertex proteins penton base and fiber, preventing release of the fiber protein and stabilizing the capsid. Loss of the vertex complex formed by penton base and fiber is presumed to be a required step in HAdV cell entry [22], [23]. In particular, dissociation of the penton base while the virus particle is in the endosome allows release of the internal viral protein VI, which is membrane lytic and can disrupt the endosomal membrane [24], [25]. The previous structural analysis of a HAdV/HD5 complex was at moderate (12 Å) resolution and led to the identification of strong HD5 binding proximal to the interface between penton base and fiber. Sequence analysis identified a negatively charged region in fiber that might form favorable interactions with HD5 and that was only present in sensitive HAdV species. Infectivity studies of virus chimeras with this fiber region swapped between sensitive and resistant HAdV species indicated that this region, together with penton base, is involved in adenoviral neutralization [13].

Here we use cryoEM and Molecular Dynamics Flexible Fitting (MDFF) simulations to further characterize the HD5 binding site at the interface between the HAdV penton base and fiber proteins. Subnanometer (8–9 Å) resolution cryoEM structures of HD5 complexed with both neutralization-sensitive and -resistant HAdV chimeras are presented. The MDFF simulations indicate that for the sensitive HAdV chimera HD5 binding is likely to involve a penton base surface loop that is predicted to be intrinsically disordered. The functions of intrinsically disordered proteins include regulation, signaling and molecular recognition, and they may serve to promote binding to multiple interaction partners [26], [27]. Their hallmark is the absence of rigid 3D structure under physiological conditions. CryoEM density is missing for the intrinsically disordered loops of the penton base. Therefore, MDFF was used to restrain the majority of the vertex model in well-defined cryoEM density while the conformation of the loops and their interaction with HD5 was dependent mainly on the potential energy function. Analysis of the stabilization effect calculated for HD5 binding at this site of the sensitive HAdV chimera provides a plausible molecular mechanism for HAdV susceptibility to defensin inactivation.

## Results

### CryoEM structures of HD5 complexed with neutralization-sensitive and -resistant HAdVs

In our previous structural study on the interaction of HD5 with a neutralization-sensitive HAdV chimera, Ad5.F35, we observed thousands of binding sites for HD5 over a significant portion of the capsid surface [13]. In particular we noted that HD5 interacts with flexible surface loops of the major capsid proteins, hexon and penton base, as well as with fiber shaft. Visualization of thousands of binding sites on the capsid is consistent with the results of an equilibrium-binding assay that showed approximately 3,000 HD5 molecules bound to each HAdV-5 virus particle [13]. HD5 binds at lower levels to resistant HAdVs, suggesting that there may be high and low affinity sites on the capsid and that HD5 binding to a subset of these sites on sensitive virus species results in neutralization. The previous structural study was performed with a relatively high (20 µM), neutralizing concentration of HD5 in order to saturate all of the possible binding sites on the virion [1], [21]. The former analysis implicated both a negatively charged region near the N-terminus of the fiber (*i.e.* 18-EDES-21 in Ad5.F35) and the penton base in HD5 mediated neutralization. In resistant HAdV types the corresponding region of the fiber is non-polar and positively charged (*i.e.* 18-GYAR-21 in HAdV-19c).

In the current study we used a lower, but still neutralizing concentration of HD5 (5 µM) with the goal of predominantly populating and visualizing individual high affinity binding sites. To this end, we determined subnanometer (<10 Å) resolution cryoEM structures of HD5 in complex with two HAdV chimeras comprised of capsid proteins from resistant and sensitive serotypes that were previously examined for susceptibility to HD5 antiviral activity [13]. Ad5.F35 contains the short-shafted HAdV-35 fiber incorporated into the HAdV-5 capsid and is sensitive to HD5 neutralization. The sensitivity of Ad5.F35 to HD5 is comparable to that of HAdV-5 and HAdV-35 [13]. Ad5.PB/GYAR, which has the HAdV-19c penton base within the HAdV-5 capsid and a mutated HAdV-5 fiber (with 18-GYAR-21), is completely resistant to HD5 activity.

**Figure 1:**
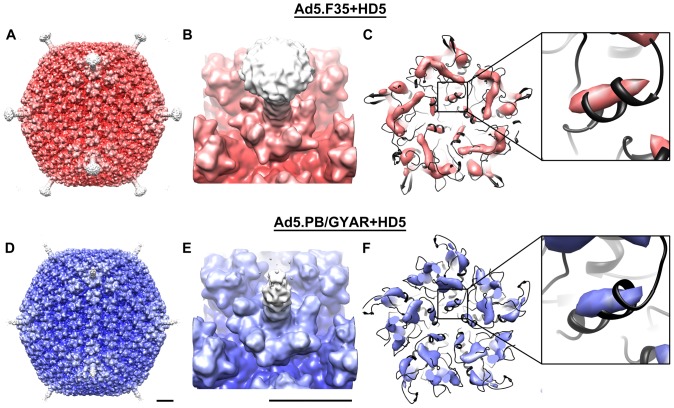
CryoEM structures of HD5 bound to neutralization-sensitive (Ad5.F35) and -resistant (Ad5.PB/GYAR) chimeric HAdVs. (A,D) Reconstructions viewed along icosahedral 2-fold axes and shown radially color-coded in red for the sensitive HAdV+HD5 complex and blue for the resistant HAdV+HD5 complex. (B,E) Enlarged views of the penton base and fiber of both HAdV+HD5 complexes. Only a portion of the Ad5.PB/GYAR fiber is reconstructed due to length (>300 Å). (C,F) Density rods are observed for penton base α-helices within both HAdV+HD5 complexes. Atomic models (black) for the HAdV-5 penton base in Ad5.F35 and the HAdV-19c penton base in Ad5.PB/GYAR are shown docked within the cryoEM density. The isosurface threshold level for the density is chosen to highlight the density rods. The enlarged insets show one density rod aligned with one α-helix confirming the subnanometer (<10 Å) resolution of the structures. Scale bars, 100 Å.

The neutralization-sensitive and -resistant HAdV chimeras were incubated in the presence of 5 µM HD5 then applied to grids and flash frozen for cryoEM analysis. This HD5 concentration is sufficient for neutralizing the sensitive HAdV chimera. Datasets were collected on an FEI Polara microscope (300 kV, FEG) under liquid nitrogen temperature. Image processing was performed as described earlier [28], and the final 3D reconstructions for Ad5.F35+HD5 and Ad5.PB/GYAR+HD5 have resolutions of 9.7 Å and 8.1 Å, respectively, by the Fourier Shell Correlation 0.5 threshold criterion (Fig. 1 and Fig. S1).

The most noticeable differences between these two virus-defensin structures lie within the vertex region, and are largely due to penton base and fiber substitutions. The short-shafted fiber is fully visible in the Ad5.F35+HD5 structure (Fig. 1A,B), whereas the longer fiber is only partially reconstructed in the Ad5.PB/GYAR+HD5 structure (Fig. 1D,E). Fitting of atomic models for the HAdV-5 and HAdV-19c penton base into our cryoEM density maps shows several α-helices from the docked atomic models that match strong regions in the cryoEM maps, indicating that the resolution for these complexes is in the subnanometer range (Fig. 1C,F and Fig. S1). Compared to the previous cryoEM study which used 20 µM HD5 [13], much less HD5 density is observed. Previously, we found that HD5 interacts with multiple flexible regions within hexon, penton base, and fiber proteins of the HAdV capsid. The strongest HD5 density observed on top of the hexons in our prior study is similar to what is observed on the hexons in both new cryoEM structures.

**Figure 2:**
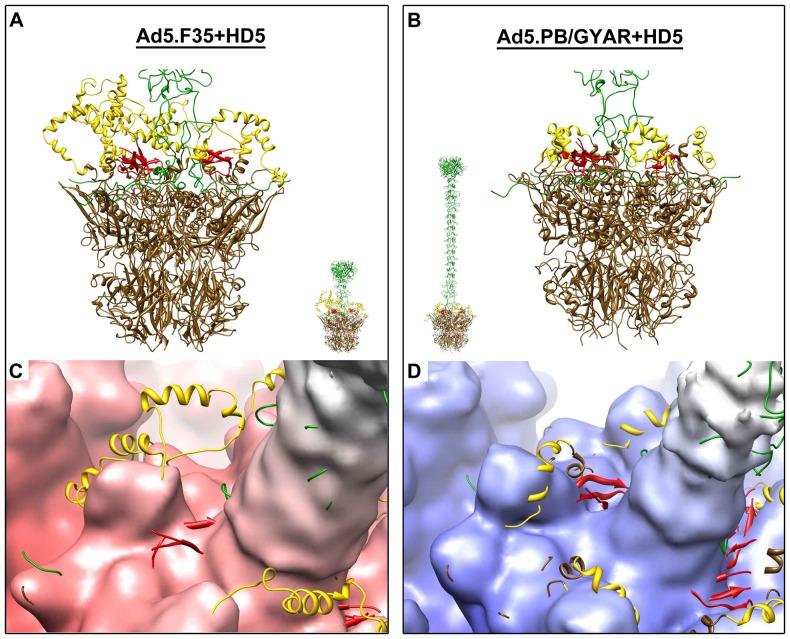
Modeling and cryoEM guided molecular dynamics simulations of the interaction between HD5 monomers and vertex proteins of the defensin-sensitive (Ad5.F35) and defensin-resistant (Ad5.PB/GYAR) HAdV chimeras. (A) Atomic model of the penton base (brown with RGD loops in yellow) and fiber (green) of Ad5.F35 with docked HD5 monomers (red). (B) Similar atomic model of Ad5.PB/GYAR. The smaller model representations in panels A and B show the full length fibers. (C,D) Atomic models of the vertex regions with HD5 monomers shown docked within the cryoEM density.

We expected to observe HD5 density at the critical site of the Ad5.F35+HD5 complex and not at the corresponding site of the Ad5.PB/GYAR+HD5 complex. After docking complete atomic models for the vertex regions into the two cryoEM density maps (Fig. 2), we realized that the density at the predicted critical HD5 binding site was weaker than expected for the defensin-sensitive Ad5.F35+HD5 complex. In the cryoEM structure of Ad5.F35+HD5 there is density above the fiber EDES sequence and next to the penton base RGD loop but it only partially covers a docked HD5 monomer (Fig. 2C). Slightly less density was observed at this site in the resistant complex (Fig. 2D). We speculate that significant disorder and flexibility at these sites would make it difficult to directly visualize HD5 density even in higher resolution structures. High resolution x-ray crystallographic and cryoEM structures of adenovirus have large regions of the penton base RGD loop missing due to disorder [29], [30] and these regions are adjacent to the critical site for HD5 neutralization proposed from the previous cryoEM and viral chimera study [13]. In addition, there is a symmetry mismatch between the pentameric penton base and the trimeric fiber that also complicates reconstruction of density in this area.

### Modeling of HD5 monomers at the HAdV vertex

In our previous study we showed that HD5 binds to HAdV capsid determinants within the vertex region at the point of contact between fiber and penton base and that disruption of these sites leads not only to resistance, but also to enhanced infection [13]. To gain insight into the structural basis for HAdV susceptibility to HD5 antiviral activity and to better define the critical neutralization site, we built molecular models for the vertex regions of Ad5.F35+HD5 and Ad5.PB/GYAR+HD5. These molecular models were refined into our cryoEM density maps using cryoEM guided molecular dynamics based flexible fitting with the MDFF software package [31]. The MDFF method allows the application of an external force based on the cryoEM density map to guide atomic models into agreement with the density while at the same time preserving correct stereochemistry by use of a standard potential energy function during the molecular dynamics simulation. The hybrid cryoEM/MDFF approach has been applied to several macromolecular complexes [32], including an engineered HAdV vector [33] and HAdV in complex with coagulation factor X (FX) [34]. In the HAdV-FX study, the hybrid approach led to determination of the molecular interaction interface between hexon and FX.

Complete atomic models were built for the penton base/fiber complexes of the Ad5.F35 and Ad5.PB/GYAR chimeras (Fig. 2A,B). The HAdV-5 penton base was constructed using the crystal structure of the HAdV-2 penton base as a template, which is 98% identical [35]. The long flexible RGD-containing loops (77aa) absent in the HAdV-2 penton base crystal structure were added into the HAdV-5 penton base homology model using the Rosetta *de novo* structure prediction protocol [36]. Five different Rosetta generated loop models were docked into the five sites of the pentameric HAdV-5 penton base. A model for the HAdV-19c penton base was obtained using the automated *ab initio* I-TASSER protein structure prediction server [37]. Fibers for both chimeras were constructed using the HAdV-2 fiber crystal structure [38] as a template. The HAdV-2 fiber crystal structure contains atomic coordinates for the trimeric fiber knob and shaft domain, which includes a repeating sequence motif. Assembly of the complete vertex models was facilitated by the crystal structure of the HAdV-2 penton base in complex with the N-terminal portion of the HAdV-2 fiber [35]. The fiber peptide in this crystal structure contains a conserved motif that binds at the interface of adjacent penton base subunits.

The crystal structure of an HD5 monomer [3] was positioned directly above the three occurrences of the fiber sequence (EDES or GYAR) that differs in the vertex models for the defensin-sensitive and defensin-resistant chimeras. Slightly more density was observed at this site in the cryoEM structure of the defensin-sensitive chimera than in the defensin-resistant chimera (Fig. 2C,D). Presumably the HD5 density at this site is weak because of both structural heterogeneity in this region and because of smearing by the imposed icosahedral symmetry during calculation of the cryoEM structures. Five-fold symmetry was imposed on the vertex regions, which is correct for the pentameric penton base but incorrect for the trimeric fiber and possibly incorrect for HD5. Thus, we are using MDFF to restrain the majority of the HAdV vertex atoms within the cryoEM density while relying more heavily on the potential energy function to position HD5 and the flexible penton base RGD-containing loops, which do not have strong cryoEM density.

**Figure 3:**
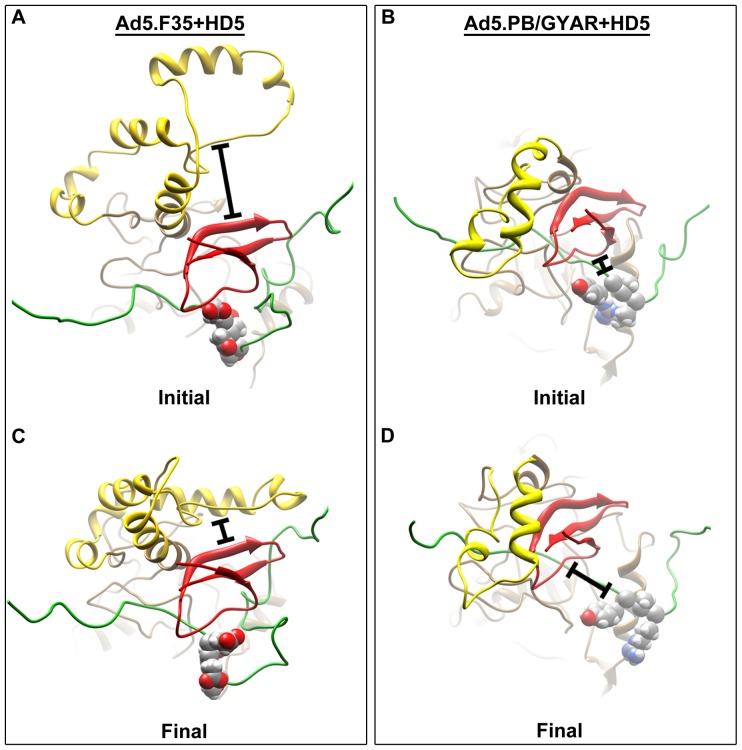
Movement of the RGD-containing loop and HD5 during the molecular dynamics simulations of the defensin-sensitive (Ad5.F35) and defensin-resistant (Ad5.PB/GYAR) HAdV chimeras. (A,C) Initial and final MDFF coordinates for one simulation of the Ad5.F35+HD5 interaction. The final coordinates show the HD5 peptide in close proximity to the fiber sequence 18-EDES-21 (spheres) and enveloped by the RGD-containing loop of the penton base. The bars indicate the extent of the movement of the RGD-containing loop toward HD5 during the simulation. (B,D) Initial and final MDFF coordinates for one simulation of the Ad5.PB/GYAR+HD5 interaction. The bars indicate the extent of the movement of HD5 away from the fiber sequence 18-GYAR-21 (spheres) during the simulation.

During MDFF simulations the HD5 monomers remained near the critical fiber sequence (18-EDES-21) in Ad5.F35 (Fig. 3 and Movie S1). Strikingly however, HD5 monomers were repelled from the corresponding fiber sequence (18-GYAR-21) of Ad5.PB/GYAR (Fig. 3 and Movie S2). Twenty-four different starting orientations of HD5 monomers were tested and in all cases the penton base RGD-containing loops of Ad5.F35 enveloped and interacted favorably with the HD5 monomers by the end of MDFF simulations. Significant movement of the Ad5.F35 RGD-containing loops was observed toward the HD5 monomers even during relatively short (100-picosecond) MDFF simulations (Fig. 3 and Movie S1). In contrast, significantly less extensive and fewer favorable interactions were found between HD5 and the RGD-containing loops of defensin-resistant Ad5.PB/GYAR (Fig. 3 and Movie S2). These observations correlate with the intermolecular nonbonded interactions calculated between the HD5 monomers and the fiber and penton base proteins at the end of the MDFF simulations (Table 1). The intermolecular nonbonded energies reported include Van der Waals and electrostatic interactions between separate polypeptide chains.

**Table 1:** Intermolecular nonbonded energies for HD5 monomers with adenovirus vertex proteins.

	Nonbonded energy between HD5 and fiber (kcal/mol)^a^	Nonbonded energy between HD5 and penton base (kcal/mol)^a^
Ad5.F35 (defensin-sensitive)	−202	−173
Ad5.PB/GYAR (defensin-resistant)	+20	−65

a Average over 24 HD5 monomer positions after MDFF simulations. Note that negative values for the nonbonded energy are favorable.

### The vertex of the defensin-sensitive HAdV accommodates HD5 dimers

**Figure 4:**
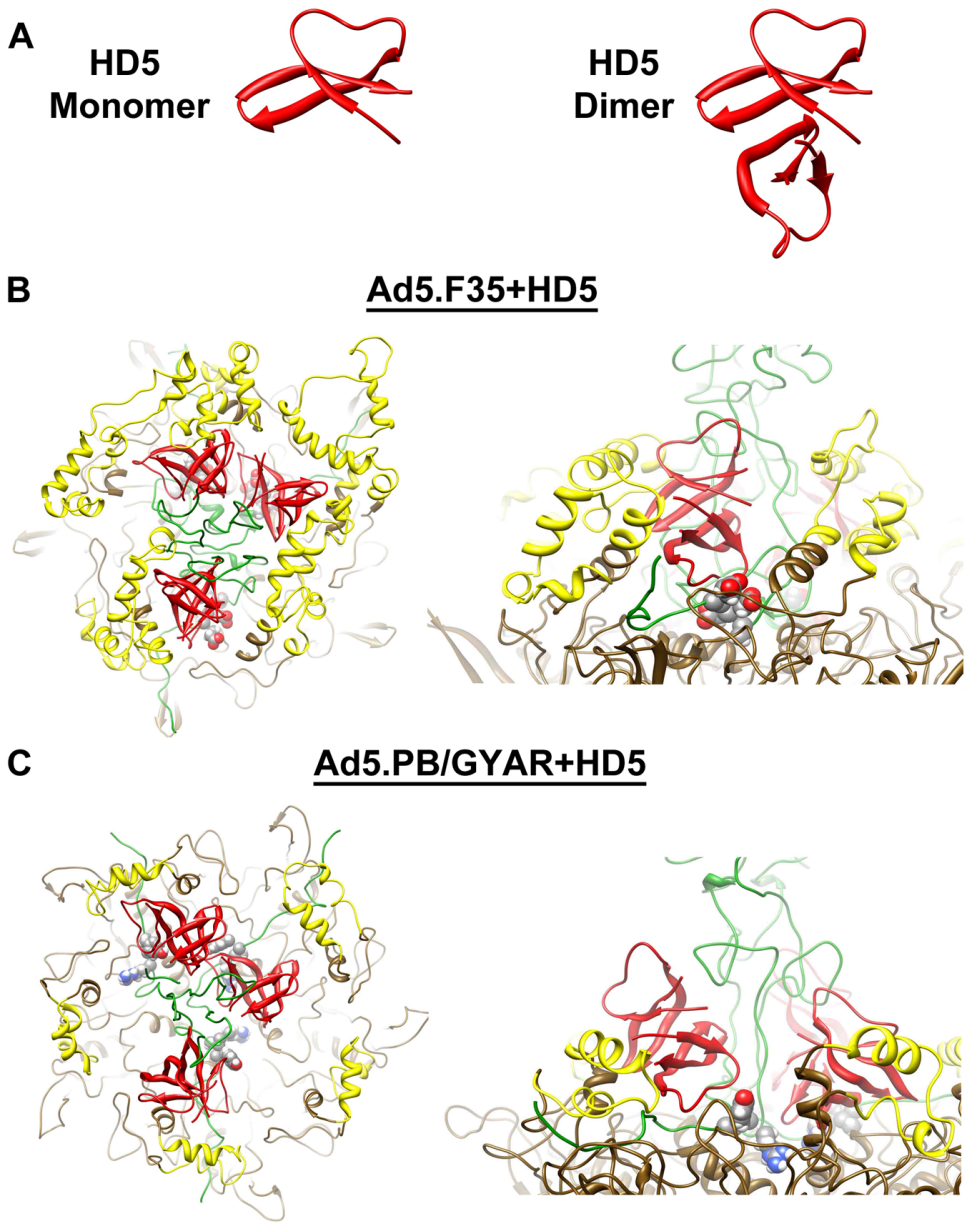
Modeling and cryoEM guided molecular dynamics simulations of the interaction between HD5 dimers and complete vertex regions of the defensin-sensitive (Ad5.F35) and defensin-resistant (Ad5.PB/GYAR) HAdV chimeras. (A) Comparison of the HD5 monomer (left) and HD5 dimer (right) structures (PDB-ID 1ZMP). (B) Final MDFF coordinates for one vertex simulation of the Ad5.F35+HD5 interaction shown in top and side views. The three HD5 dimers are each in close proximity to the fiber sequence 18-EDES-21 (spheres) and flanked by the RGD-containing loops of the penton base. (C) Final MDFF coordinates for one vertex simulation of the Ad5.PB/GYAR+HD5 interaction. The HD5 dimers are less closely associated with the fiber sequence 18-GYAR-21 (spheres) and less well enveloped by the RGD-containing loops compared to the defensin-sensitive vertex. The coloring scheme is as in Figure 2.

In crystal structures α-defensins are found as dimers (Fig. 4A) [2], [3]. Recent studies indicate the importance of dimerization for viral neutralization activity, as an obligate monomer of HD5 was able to bind to the HAdV-5 capsid but was non-neutralizing [6]. Therefore, we repeated the molecular dynamics simulations with HD5 dimers modeled at the vertex sites within Ad5.F35 and Ad5.PB/GYAR. In the case of Ad5.F35, the fiber/penton base interface easily accommodated HD5 dimers (Fig. 4B). In fact, the presence of HD5 dimers rather than monomers leads to even more favorable intermolecular nonbonded interactions between HD5 and the HAdV vertex proteins than observed for HD5 monomers (Table 2). In the case of Ad5.PB/GYAR, the short penton base RGD-containing loops are not able to envelop the HD5 dimers nearly as well as Ad5.F35 (Fig. 4C), and significantly less favorable interactions are modeled.

**Table 2:** Intermolecular nonbonded energies for HD5 dimers with adenovirus vertex proteins.

	Nonbonded energy between HD5 and fiber (kcal/mol)^a^	Nonbonded energy between HD5 and penton base (kcal/mol)^a^
Ad5.F35 (defensin-sensitive)	−274	−308
Ad5.PB/GYAR (defensin-resistant)	−90	−85

a Average over 24 HD5 dimer positions after MDFF simulations.

### Intrinsic disorder at the HD5 binding site

In total twenty-four different starting orientations of HD5 monomers and twenty-four orientations of HD5 dimers were simulated for the defensin-sensitive and defensin-resistant HAdV chimeras. In one simulation run, three different HD5 orientations were modeled with a complete vertex formed by a pentameric penton base and a trimeric fiber. Since the RGD-containing loops of penton base are known to be highly flexible, and numerous feasible loop models were generated by Rosetta, we built a complete penton base with five different initial RGD-containing loop models. We reasoned that it would be more realistic to use five different loop models than to pick one loop model arbitrarily and use it at all five sites within the pentamer. Several factors led to our decision to perform MDFF simulations with HD5 in multiple starting orientations. These factors included the observation of weak and five-fold symmetrized defensin density in the vicinity of the critical binding site, which precluded initial docking of HD5 coordinates on the basis of HD5 shape within the cryoEM density (Fig. 2C,D). In addition, the nature of HD5 with positive charge on multiple surfaces of the monomer and dimer made it possible to orient the peptide in multiple reasonable starting orientations with respect to the negatively-charged 18-EDES-21 sequence of the Ad5.F35 fiber.

One interesting finding that emerged from the MDFF simulations is that the critical binding site between the fiber N-terminal region and the penton base RGD-containing loops seems to be highly structurally malleable. This site in the defensin-sensitive HAdV chimera can form various favorable binding pockets for HD5. This led us to submit the HAdV5 and HAdV19c penton base protein sequences to the PrDOS ProteinDisOrder System prediction webserver [39]. The HAdV5 penton base is predicted to have a large disordered region (aa 297–373) (Fig. S2), which corresponds well to the flexible RGD loop region (aa 297–374) as defined by sequence alignment to the HAdV2 penton base crystal structure [35]. The HAdV19c penton base also is predicted to have a disordered region (aa 294–316), but it is significantly shorter (Fig. S2). The MDFF simulations show that the disordered regions of both penton bases interact with HD5. Regardless of the starting orientations for the HD5 monomer/dimer, or the conformation of the RGD-containing loops, after the simulation we observed favorable intermolecular nonbonded interactions between HD5 and penton base and also between HD5 and fiber. Simulations with the defensin-resistant HAdV chimera showed that although favorable interactions could be found, they were less favorable overall than for the defensin-sensitive chimera and often only between HD5 and either penton base or fiber but not both. This implies that although HD5 may bind to the vertex region of the defensin-resistant chimera it might not bridge the penton base and fiber and stabilize the vertex as it appears to do for the sensitive chimera.

**Figure 5:**
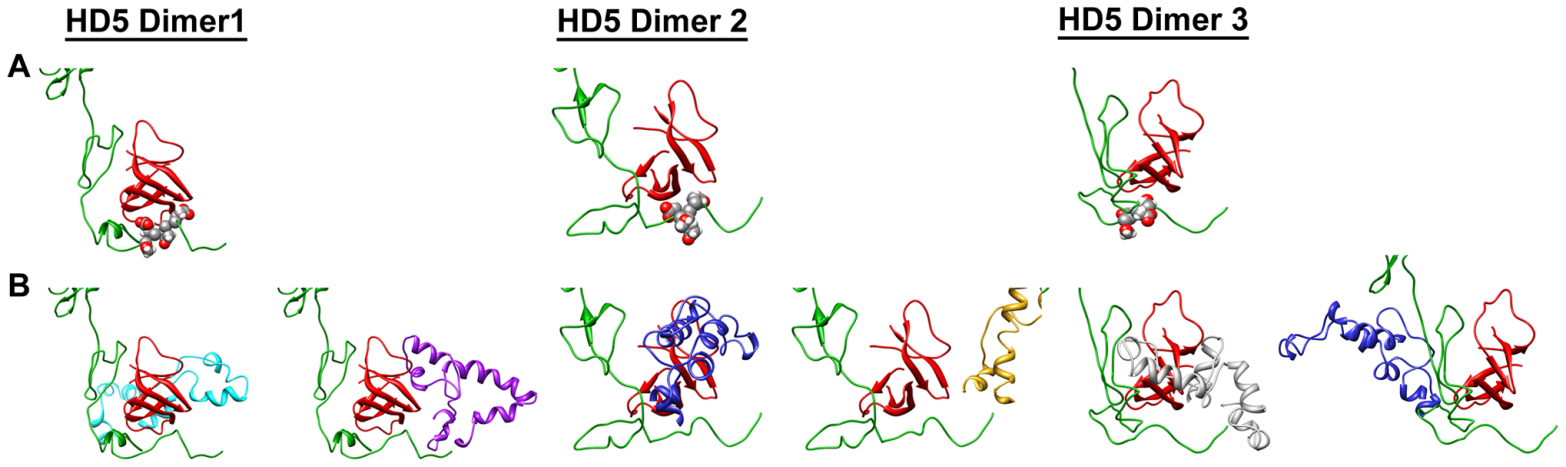
Structural malleability of the binding pocket within the defensin-sensitive HAdV chimera (Ad5.F35). (A) Comparison of three different HD5 dimer orientations each with respect to one fiber subunit containing the closest 18-EDES-21 sequence (spheres). (B) Comparison of penton base RGD-containing loop interactions with the same three HD5 dimers. The penton base loops are shown without the rest of the penton base. The models in panels A and B are all based on final MDFF coordinates from one vertex with a trimeric fiber and a pentameric penton base. The five penton base loops are shown in purple, cyan, blue, gold, and gray and the rest of the coloring scheme is as in Figure 2.

The structural malleability of the binding pocket within the defensin-sensitive HAdV chimera is illustrated in Figure 5 for one vertex region. Three HD5 dimers were started in different orientations and in close proximity to the N-terminal fiber residues 18-EDES-21. At the end of the MDFF simulation all three HD5 dimers remained in close proximity to the EDES sequence, but they were oriented differently with respect to the fiber shaft and formed diverse interactions with loops of the fiber shaft (Fig. 5A). The intermolecular nonbonded energies reported in Table 3 indicate that each of these three HD5 dimers formed favorable interactions with one or more of the fiber subunits at the vertex. The 3-fold β-spiral repeat elements comprising the fiber shaft [38] facilitate the interaction with multiple HD5 dimers.

**Table 3:** Intermolecular nonbonded energies for three HD5 dimers with each of the subunits of fiber and penton base at one defensin-sensitive adenovirus vertex.^a^

	HD5 Dimer 1	HD5 Dimer 2	HD5 Dimer 3
**Fiber**	−171	−176	−12
**Fiber**	−4	−85	−42
**Fiber**	−241	+9	−225
**Penton Base**	−272	−25	0
**Penton Base**	−235	+22	0
**Penton Base**	0	−50	−3
**Penton Base**	0	−178	−109
**Penton Base**	−8	0	−187
**Sum**	**−931**	**−483**	**−578**

a Nonbonded energies reported in kcal/mol.

Similarly, the three HD5 dimers of this same vertex formed multiple and extensive interactions with the intrinsically disordered RGD-containing loops of the penton base (Fig. 5B). Although all of the loops moved toward the HD5 dimers and interacted favorably with HD5 by the end of the MDFF simulations, there did not appear to be a single preferred mode of interaction. Rather, the relatively long length of the loop (77aa) and its conformational flexibility resulted in different, yet highly favorable, interactions with HD5. This was true regardless of the starting orientation of HD5 in all twenty-four simulations of dimers within defensin-sensitive binding pockets. As indicated by the intermolecular nonbonded energies for one vertex (Table 3), each loop interacts with more than one HD5 dimer and each HD5 dimer interacts favorably with one or two loops.

### Stabilization of the defensin-sensitive HAdV vertex region by HD5

A striking result from the MDFF simulations is that HD5 dimers bound to the vertex region substantially stabilize the complex of penton base and fiber. If the working model for HAdV cell entry is correct, stabilizing the non-covalent association between fiber and penton base could block subsequent capsid uncoating steps that are necessary for escape of the virion from the endosome and a productive infection. The total nonbonded interaction energy calculated for three HD5 dimers at the vertex shown in Figure 5 corresponds to a stabilization of 1,992 kcal/mol. For comparison, the average nonbonded energy calculated between fiber and penton base indicates a more modest favorable interaction of 670 kcal/mol. To help put these MDDF calculated potential energy values in perspective, we note that the stabilization achieved upon maturation of the bacteriophage HK97 capsid has been measured by differential scanning calorimetry to be on the order of 1,930 kcal/mol [40]. This enormous effect includes both expansion and crosslinking of the capsid proteins.

The MDFF simulations for the vertex shown in Figure 5 indicate that three HD5 dimers can provide an increase in stabilization by a factor of 3. Calculations for seven other vertices, corresponding to twenty-one additional HD5 dimer orientations, show similar stabilization increases on the order of factors of 2 to 3. The MDFF simulations presented here suggest that HD5 dimers stabilize the HAdV vertex by bridging the fiber and penton base components at the critical fiber site. This stabilization effect could potentially lock the capsid in a structure that prevents release of the membrane lytic protein VI and therefore restricts viral escape from the endosome.

## Discussion

The simulations presented here indicate that an intrinsically disordered loop of the adenovirus capsid can interact with HD5 favorably in multiple different ways. Given the strong nonbonded interaction energies calculated for the Ad5.F35/HD5 interaction, it is possible that once HD5 is bound at the vertex sites it is not released. In fact, it has been noted that a protein with intrinsic disorder can bind permanently with an interaction partner [26]. If HD5 bound at the vertex sites of sensitive HAdV types can permanently lock the vertex region, this would presumably block release of the membrane lytic factor in the endosome and consequently block cell entry. Consistent with this, recent studies have shown that HD5 selectively increases the tensile strength of the capsid vertex region [41]. We built a space filling representation of one Ad5.F35 vertex with three bound HD5 dimers (Fig. S3) and we find that several of the RGD sites are still accessible on the surface. This is consistent with the experimental finding that the presence of HD5 does not block virus internalization [21].

This study presents detailed models for the interaction of a human α-defensin, HD5, with two HAdV chimeras, one of which is sensitive to defensin antiviral activity and one of which is resistant. Previously, a charged stretch of four residues within the N-terminal region of fiber was implicated as playing a role in the critical binding site for HD5 [13]. This site is at a position of symmetry mismatch within the HAdV capsid, where the trimeric fiber interacts with the pentameric penton base, and is at least partially flexible [35]. In addition, this site is next to the flexible RGD-containing loop of the penton base. These factors made it difficult to directly resolve density for HD5 in the current structures, although we noted slightly more density attributable to HD5 in this site for the sensitive chimera than in the resistant chimera. In order to overcome this limitation we used a hybrid approach including MDFF to model the interaction of HD5 with both HAdV capsids. In this case we are relying heavily on the MDFF potential energy functions to model the interactions between HD5 and the intrinsically disordered region of penton base, as strong cryoEM density is not observed for these regions. The simulations reveal that HD5 bridges the penton base and fiber and thus stabilizes the vertex complex formed by these two proteins within the sensitive chimera. The bridging interactions involve the negatively charged EDES sequence in the N-terminal fiber region, which is of opposite charge in the resistant chimera, and multiple and varying residues of the penton base RGD loop.

The MDFF simulations show the critical binding pocket to have a large degree of malleability, as expected for an intrinsically disordered region. The RGD-containing penton base loops of the sensitive HAdV chimera are observed in the simulations to envelop HD5 monomers and dimers at the top of the penton base near the critical fiber sequence. We speculate that defensin neutralization of HAdV may be taking advantage of the inherent flexibility of the RGD-containing loops. Flexibility of these loops is thought to be important for their interaction with αv integrins during cell entry [22]. The simulations also indicate multiple favorable orientations for HD5 dimers at the interface between penton base and fiber. This variability within the atomic models for the HD5 interaction with the HAdV vertex is consistent with difficulty we had in observing density for HD5 at the vertex in the cryoEM structure of the defensin-sensitive chimera.

A recent study investigating the critical determinants of HD5 activity against HAdV found that HD5-mediated neutralization depends upon specific binding interactions to the viral capsid mediated in part by critical arginine residues, R9 and R28, in HD5 [6]. Visual inspection of the MDFF results for HD5 dimers bound to HAdV vertex proteins indicates that the critical arginines contribute to the calculated intermolecular nonbonded energies. Gounder *et al.* [6] also found that stabilization of the HD5 dimer is critical for neutralization of HAdV. The MDFF-based finding of more favorable interaction energies for HD5 dimers than for HD5 monomers with the defensin-sensitive HAdV chimera is consistent with this experimental result. We speculate that higher order multimerization of HD5 above the vertex region would provide further stabilization. The malleability for the critical binding pocket and the long, flexible RGD-containing penton base loops of the sensitive HAdV chimera should allow for various types of HD5 associations with the penton base/fiber complex. It is likely that additional HD5 interactions at the vertex would stabilize the protein complex and would impede its timely dissociation in the endosome.

The average nonbonded interaction energy calculated at the end of the MDFF simulations for one HD5 dimer with a penton base/fiber complex indicates a stabilization of 582 kcal/mol. This is on the order of the nonbonded interaction energy calculated between penton base and fiber at one vertex, which indicates a similar stabilization of 670 kcal/mol. Therefore, if three HD5 dimers bind at the same vertex, the overall increase in stabilization of the vertex is on the order of a factor of three. Calculation of a significant stabilization effect on the vertex by HD5 is consistent with the experimental results from a thermostability assay showing that HD5 stabilizes the defensin-sensitive HAdV5 capsid [21]. In the presence of HD5, the release of fiber and membrane lytic protein VI was shifted from ∼49°C to 61° and 67°C respectively. This thermostability assay is designed to mimic virus disassembly in the endosome [25]. Our working model for HD5 neutralization of HAdV is that binding of defensin at critical sites on the HAdV capsid could prevent dissociation of fiber from penton base, which is thought to be an early step in HAdV disassembly during cell entry. Stabilization of the association of fiber with the penton base could in turn block subsequent steps in uncoating that lead to the release of protein VI and disruption of the endosomal membrane [13]. The ability of HD5 to block viral uncoating during cell entry has been demonstrated [42]

The cryoEM structures presented here were determined with 5 µM HD5, which is close to the IC50 for inhibition of HAdV-5 infection of 3–4 µM [21]. The concentration of HD5 within small localized areas of the intestine has been estimated as 100 to 1000 times higher [1]. If the model for defensin neutralization of HAdV is correct, then we assume that HD5 must bind and stabilize all twelve vertices of the HAdV capsid in order to completely block infectivity. Given the variability we observed in the MDFF simulations for the interaction of HD5 with the HAdV vertex proteins, it is possible that at low µM concentrations of HD5 most but not all of the vertices are highly stabilized by defensin. We speculate that if a few vertices are not stabilized by HD5, this could allow partial disassembly of the capsid and release of protein VI in the endosome. This would be consistent with measured low levels of infectivity for multiple sensitive HAdV types in the presence of 15 µM HD5 [13].

In summary, our goal was to gain more precise structural information of the interaction of HD5 with a defensin-sensitive and a resistant HAdV chimera. Due to the intrinsic disorder of the RGD-containing penton base loops of the sensitive chimera we relied on molecular dynamics simulations to enhance the model of defensin interaction. The simulations indicate that HD5 dimers can stabilize the interaction between penton base and fiber, but only in the context of the sensitive chimera. A high degree of conformational flexibility was found for the critical binding pocket resulting in a variety of stabilizing interactions between defensin and the capsid vertex proteins. Based on our structural analysis and modeling studies, it is likely that intrinsic disorder is an important aspect of the binding pocket that contributes to the susceptibility of a particular HAdV types to defensin neutralization.

## Materials and Methods

### CryoEM and Image Processing

Samples of two HAdV chimeras, Ad5.F35 and Ad5.PB/GYAR, were prepared as previously described [13]. Purified virus (160 µg/ml) was combined with HD5 (5 µM) and incubated on ice for 45 minutes. These samples were then applied to Quantifoil grids and rapidly frozen in liquid ethane using a homebuilt vitrification device. For high resolution cryoEM data acquisition, electron micrographs were collected on an FEI Polara (300 kV, FEG) operated at liquid nitrogen temperature. A Gatan UltraScan 4000 CCD camera was used for recording images at an absolute magnification of 397,878×. The underfocus values of the micrographs ranged from 0.5 µm to 4 µm. Datasets were collected for HD5 complexed with Ad5.F35 and Ad5.PB/GYAR that included 3,515 and 3,620 particle images, respectively. Individual particles were manually selected and centered using in-house scripts and stacks were generated at various pixel sizes (4.5 Å, 2.2 Å, and 1.5 Å) suitable for single particle image processing. Particle images with a coarser image size were used at the beginning of refinement. Initial defocus and astigmatism estimates were determined using the program CTFFIND3 [43]. A cryoEM structure of Ad5.F35 [28] was used as the starting model for FREALIGN refinement [44]. Intermediate refinement rounds were performed using particle images with a 2.2 Å pixel size and the final refinement rounds with a particle image pixel size of 1.5 Å. For both datasets, the orientational and CTF parameters, as well as absolute magnification were refined using FREALIGN. The resolutions of the final cryoEM structures of the Ad5.F35/HD5 complex and Ad5.PB/GYAR/HD5 complex were at 9.7 Å and 8.1 Å, respectively, as measured at the Fourier Shell Correlation 0.5 threshold (Fig. S1).

### Atomic Model Building

We constructed atomic models for the vertex regions of Ad5.F35 and Ad5.PB/GYAR, which include the capsid proteins penton base and fiber. Homology models of HAdV-5 and HAdV-19c penton base proteins were generated using the HAdV-2 penton base crystal structure [35]. For Ad5.F35, the HAdV-2 penton base crystal structure (98% identity) was mutated to match the HAdV-5 penton base sequence using the amino acid substitution command available within the UCSF Chimera software package [45]. Additionally, the long flexible RGD containing loops (77aa) that are absent in the crystal structure were incorporated into the HAdV-5 penton base homology model using the Rosetta *de novo* structure prediction protocol [36]. The five RGD containing loops that were attached to the penton base model have different conformations. A homology model of the Ad19c penton base was created using the I-TASSER server for protein 3D structure prediction [37], [46]. Atomic models were created for the short-shafted fiber present in Ad5.F35 and the longer HAdV-5 fiber present in Ad5.PBGYAR based on the Ad2 fiber crystal structure [38]. The HAdV-2 fiber crystal structure contains atomic coordinates for the trimeric fiber knob and shaft domain, which includes a repeating sequence motif. Attachment of the fibers to penton base proteins was guided by the crystal structure of HAdV-2 penton base in complex with a portion of the fiber [35]. The HAdV-2 N-terminal fiber peptide includes is a highly conserved fiber motif that binds at the interface of adjacent penton base subunits.

### Molecular Dynamics Flexible Fitting

MDFF is a cryoEM guided molecular dynamics method that will flexibly fit atomic models into cryoEM density maps [31], [47]. In MDFF simulations, forces proportional to the gradient of the density map are applied to all atoms, driving the models to occupy regions of high density. Structural restraints are applied to preserve secondary structural elements so that over-fitting does not occur during the simulation. The simulations were carried out in such a way that only secondary structural elements were guided into density with a g-scale value of 0.3. We purposely kept the g-scale value rather low so that the coordinates for HD5 and the flexible penton base loops would not be forced into cryoEM density. All MDFF simulations were carried out using NAMD 2.8 [48] and the CHARMM27 force field. CryoEM density of the vertex regions was extracted from both the Ad5.F35+HD5 (defensin-sensitive) and Ad5.PB/GYAR+HD5 (defensin-resistant) maps and vertex models were docked into the selected densities using rigid body fitting in UCSF Chimera [45]. The docked coordinates served as input for 100-picosecond MDFF implicit solvent simulations. Monomeric and dimeric forms of HD5 were tested with both the defensin-sensitive and defensin-resistant vertex regions. Nonbonded energies were measured between specific polypeptide chains using the NAMD Energy plugin in VMD [49]. The MDFF simulations were performed on the Case Western Reserve University High-Performance Computing Cluster.

## Supporting Information

Figure S1 **Subnanometer resolution of cryoEM structures of HD5 bound to neutralization-sensitive (Ad5.F35) and -resistant (Ad5.PB/GYAR) chimeric HAdVs.** (A,C) Density rods are observed for hexon α-helices within both HAdV+HD5 complexes. Atomic models (black) for the HAdV-5 hexon in Ad5.F35 and the HAdV-19c penton base in Ad5.PB/GYAR are shown docked within the cryoEM density. The isosurface threshold level for the density is chosen to highlight the density rods. (B,D) Fourier shell correlation plots indicating 9.7 Å resolution for Ad5.F35+HD5 and 8.1 Å resolution for Ad5.PB/GYAR+HD5 at the FSC 0.5 thresholds.
(TIF)

Figure S2 **Prediction of intrinsically disordered regions within the HAdV5 and HAdV19c penton base proteins by the PrDOS webserver** [**39**] **.** (A) Prediction for the HAdV5 penton base of the Ad5.F35 virus chimera. A long intrinsically disordered region is found between residues 297 and 373. This corresponds well to the RGD loop (aa297–374) as assigned based on sequence alignment to the flexible residues in the HAdV2 penton base crystal structure [35]. (B) Prediction for the HAdV19c penton base of the Ad5.PB/GYAR virus chimera. A short intrinsically disordered region is found between residues 294 and 316, overlapping with the RGD loop (aa290–318). Residues above the 0.5 threshold line in these plots are predicted to be disordered.
(TIF)

Figure S3 **Space filling representation of the vertex region of Ad5.F35 with three bound HD5 dimers.** The penton base is shown mostly in brown with the RGD loops in yellow and the RGD residues in blue. The fiber is shown in green and HD5 in red. This representation was generated with the UCSF Chimera molmap command with a 5 Å resolution filter applied to the final MDFF coordinates for one vertex.
(TIF)

Movie S1 **MDFF simulation of defensin-sensitive HAdV chimera, Ad5.F35, in complex with defensin.** The movie starts with coordinates for penton base (gold and white) and fiber (green) with three docked defensin monomers (red) surrounded by five hexon trimers (blue) at one adenovirus vertex. The cryoEM structure of the complex (magenta) is faded in and the view is changed to show the whole virion. In the final segment, MDFF frames from a 100-picosecond simulation show the flexible RGD-containing penton base loops (white) envelop the defensin molecules. The last frame shows the negatively charged fiber sequence (18-EDES-21), which is critical for defensin neutralization, in a sphere representation.
(WMV)

Movie S2 **MDFF simulation of defensin-resistant HAdV chimera, Ad5.PB/GYAR, in complex with defensin.** MDFF frames from a 100-picosecond simulation show the short RGD-containing penton base loops (white) do not envelop the defensin molecules as extensively as observed for the defensin-sensitive HAdV chimera. The last frame shows the hydrophobic and positively charged fiber sequence (18-GYAR-21), which is correlated with defensin resistance, in a sphere representation.
(WMV)
