# Supplementary figures and images for: An Intrinsically Disordered Region of the Adenovirus Capsid Is Implicated in Neutralization by Human Alpha Defensin 5
Source: PLoS One. 2013 Apr 19;8(4):e61571. doi: 10.1371/journal.pone.0061571 (PMC3631211; doi:10.1371/journal.pone.0061571)

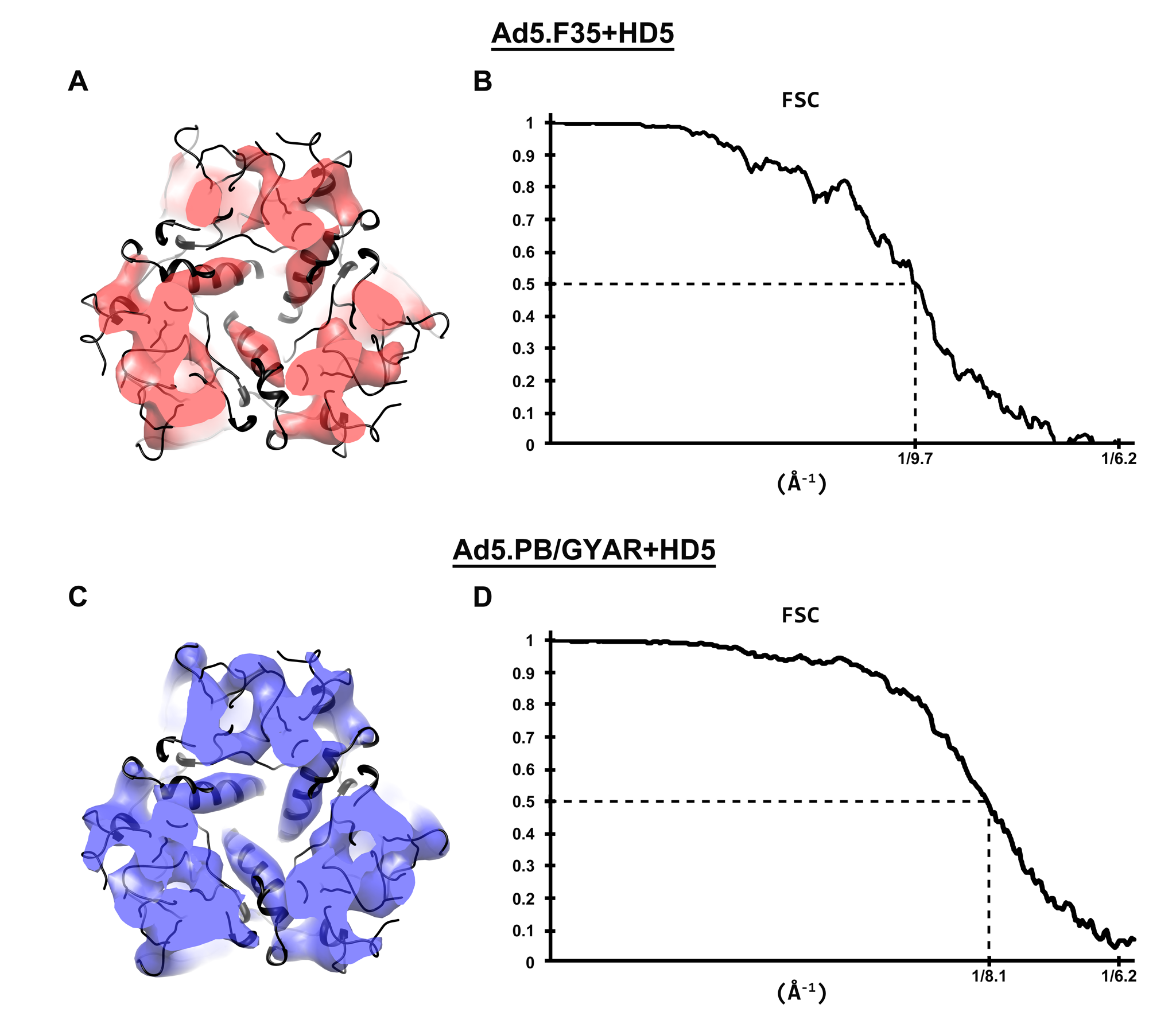

Supplement: Figure S1 — Subnanometer resolution of cryoEM structures of HD5 bound to neutralization-sensitive (Ad5.F35) and -resistant (Ad5.PB/GYAR) chimeric HAdVs. (A,C) Density rods are observed for hexon α-helices within both HAdV+HD5 complexes. Atomic models (black) for the HAdV-5 hexon in Ad5.F35 and the HAdV-19c penton base in Ad5.PB/GYAR are shown docked within the cryoEM density. The isosurface threshold level for the density is chosen to highlight the density rods. (B,D) Fourier shell correlation plots indicating 9.7 Å resolution for Ad5.F35+HD5 and 8.1 Å resolution for Ad5.PB/GYAR+HD5 at the FSC 0.5 thresholds. (TIF) [file pone.0061571.s001.tif]

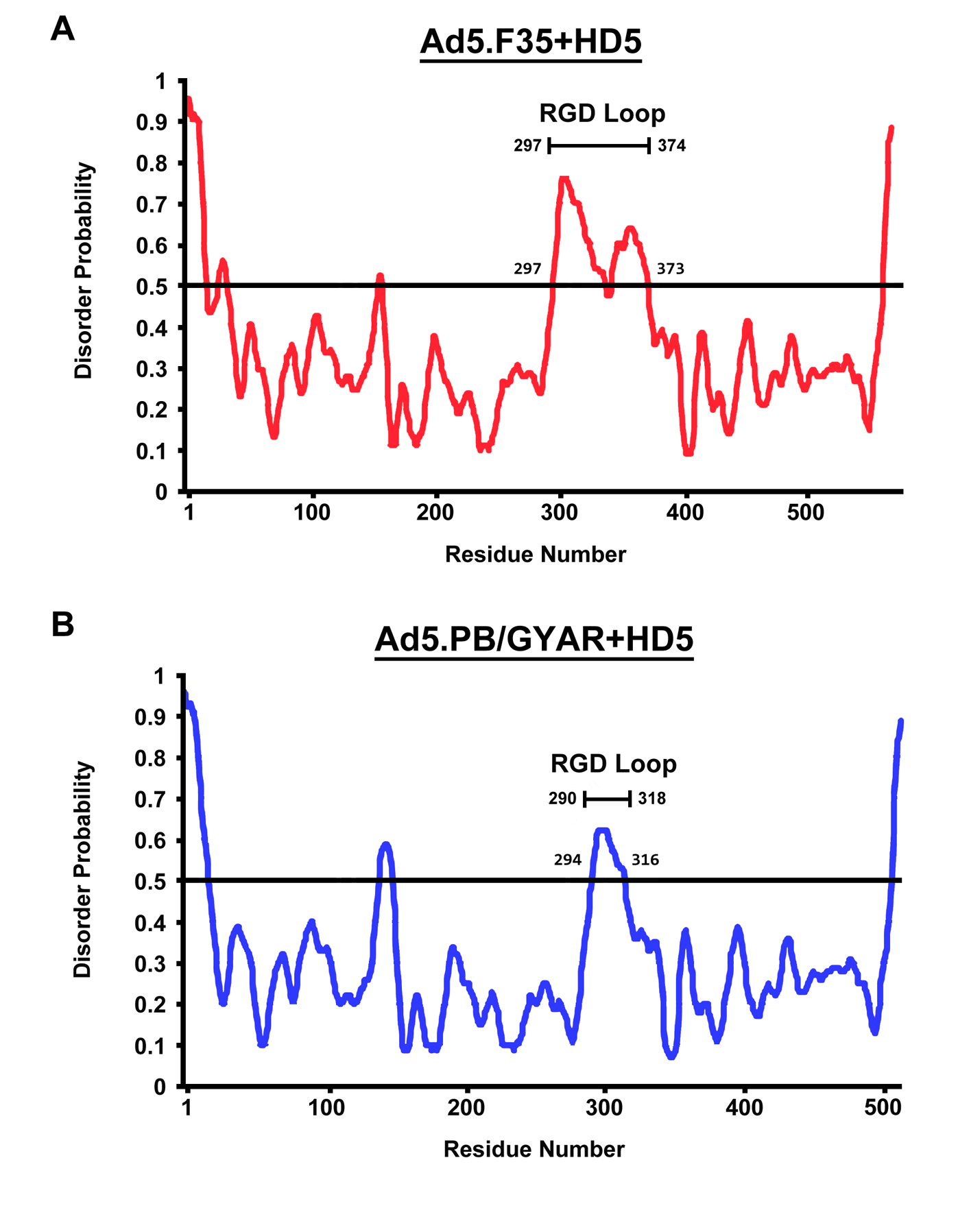

Supplement: Figure S2 — Prediction of intrinsically disordered regions within the HAdV5 and HAdV19c penton base proteins by the PrDOS webserver [39] . (A) Prediction for the HAdV5 penton base of the Ad5.F35 virus chimera. A long intrinsically disordered region is found between residues 297 and 373. This corresponds well to the RGD loop (aa297–374) as assigned based on sequence alignment to the flexible residues in the HAdV2 penton base crystal structure [35]. (B) Prediction for the HAdV19c penton base of the Ad5.PB/GYAR virus chimera. A short intrinsically disordered region is found between residues 294 and 316, overlapping with the RGD loop (aa290–318). Residues above the 0.5 threshold line in these plots are predicted to be disordered. (TIF) [file pone.0061571.s002.tif]

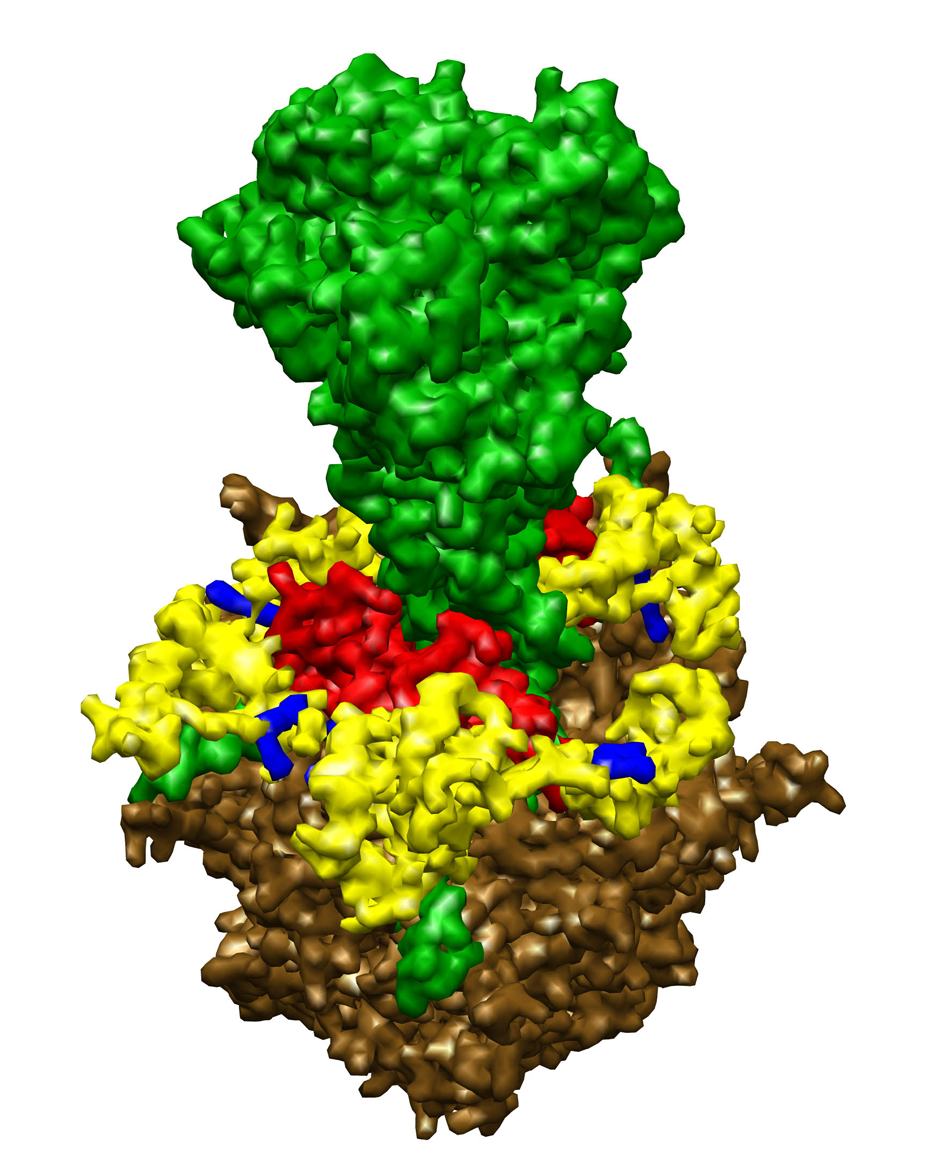

Supplement: Figure S3 — Space filling representation of the vertex region of Ad5.F35 with three bound HD5 dimers. The penton base is shown mostly in brown with the RGD loops in yellow and the RGD residues in blue. The fiber is shown in green and HD5 in red. This representation was generated with the UCSF Chimera molmap command with a 5 Å resolution filter applied to the final MDFF coordinates for one vertex. (TIF) [file pone.0061571.s003.tif]
